# New Model of Ventral Spinal Cord Lesion Induced by Balloon Compression in Rats

**DOI:** 10.3390/biomedicines8110477

**Published:** 2020-11-05

**Authors:** Petr Krupa, Katerina Stepankova, Jessica CF. Kwok, James W. Fawcett, Veronika Cimermanova, Pavla Jendelova, Lucia Machova Urdzikova

**Affiliations:** 1 Institute of Experimental Medicine, Czech Academy of Sciences, Vídeňská, 1083 Prague, Czech Republic; petr.krupa@iem.cas.cz (P.K.); katerina.stepankova@iem.cas.cz (K.S.); J.Kwok@leeds.ac.uk (J.C.K.); jf108@cam.ac.uk (J.W.F.); veronika.cimermanova@iem.cas.cz (V.C.); 2 Department of Neurosurgery, Charles University, Medical Faculty and University Hospital Hradec Králové, Sokolska 581, 50005 Hradec Kralove, Czech Republic; 3 Department of Neuroscience, Charles University, Second Faculty of Medicine, 15006 Prague, Czech Republic; 4 Faculty of Biological Sciences, University of Leeds, Leeds LS2 9JT, UK; 5 John van Geest Centre for Brain Repair, Dept. Clinical Neurosciences, University of Cambridge, Cambridge CB2 0PY, UK

**Keywords:** ventral spinal cord injury, ischemic compression injury, spinal tissue loss, astrogliosis, motoneurons

## Abstract

Despite the variety of experimental models of spinal cord injury (SCI) currently used, the model of the ventral compression cord injury, which is commonly seen in humans, is very limited. Ventral balloon compression injury reflects the common anatomical mechanism of a human lesion and has the advantage of grading the injury severity by controlling the inflated volume of the balloon. In this study, ventral compression of the SCI was performed by the anterior epidural placement of the balloon of a 2F Fogarty’s catheter, via laminectomy, at the level of T10. The balloon was rapidly inflated with 10 or 15 μL of saline and rested in situ for 5 min. The severity of the lesion was assessed by behavioral and immunohistochemical tests. Compression with the volume of 15 μL resulted in severe motor and sensory deficits represented by the complete inability to move across a horizontal ladder, a final Basso, Beattie and Bresnahan (BBB) score of 7.4 and a decreased withdrawal time in the plantar test (11.6 s). Histology and immunohistochemistry revealed a significant loss of white and gray matter with a loss of motoneuron, and an increased size of astrogliosis. An inflation volume of 10 μL resulted in a mild transient deficit. There are no other balloon compression models of ventral spinal cord injury. This study provided and validated a novel, easily replicable model of the ventral compression SCI, introduced by an inflated balloon of Fogarty´s catheter. For a severe incomplete deficit, an inflated volume should be maintained at 15 μL.

## 1. Introduction

Spinal cord injury (SCI) is a life-changing medical condition with very high rates of morbidity. There are more than 12,500 new cases of SCI each year in North America and 90% of them result from trauma caused by, for example, traffic accidents, falls from heights or sporting activities [1]. The clinical outcome depends on the severity and spinal level. The clinical treatment of severe SCI remains unsatisfactory, leaving patients with various degrees of disability and dependency. However, with recent advancements in patient care, SCI patients have good long-term survival rates leading to high lifetime costs.

Preclinical research focused on the various types of SCI has confirmed the promising, beneficial effects of immunomodulatory therapy [2,3,4,5,6], stem cells [7,8,9,10,11], trophic factors [12,13,14,15,16], electric stimulation [17,18,19], axonal regeneration and plasticity; all with the potential to enable an improved functional outcome. In the laboratory setting, several models of SCI are used. Most studies are performed on small animals, especially on rats, which exhibit similar morphological, electrophysiological and functional outcomes when compared to humans [20].

**Figure 1:**
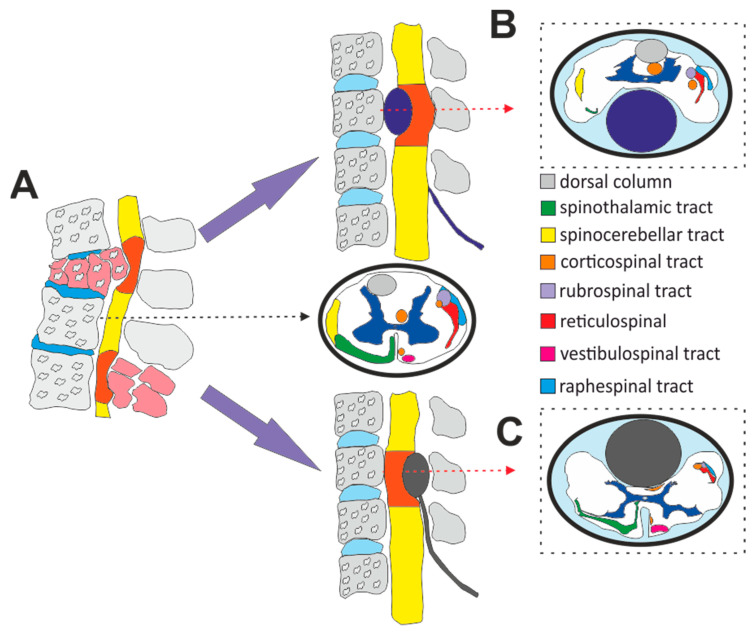
Illustrative image of ventral and dorsal compressive spinal cord lesions with a cross section of the uninjured spinal cord in a rat model (**A**). Inflation of the ventrally placed balloon mainly induces a lesion of the spinothalamic, vestibulospinal and ventral portion of the corticospinal tract (**B**). Classical placement of the catheter into the dorsal epidural space affects, in the first line, the dorsal portion of the corticospinal tract and the axons from dorsal columns (**C**). Black and red arrows represent the level of the cross sections.

The most common primary mechanism of spinal cord injury is axial loading with spinal cord compression or contusion [21]. The compression of the cord can be caused by damage to the posterior structures of the spinal column including the vertebral arch and ligaments or, more often, herniation of the intervertebral disc or vertebral body fragments into the spinal canal causing the antero-posterior narrowing of the vertebral canal (Figure 1A) [22,23]. Therefore, contusion and compression experimental models of SCI are considered to be the most relevant models. Moreover, they provide spinal cord lesions with a graded scale of severity according to the strength of the impact. Contusion injury is typically modeled using a weight drop device or impactor, affecting the dorsally exposed spinal cord [20,24,25]. Epidural compression using the Fogarty catheter was first described by Vanicky et al. in 2001 [26]. The SCI was introduced by dorsal epidural placement of the rapidly inflated balloon with 15 μL of saline, leading to a consistent severe neurological deficit. A neurological deficit correlates with heavy damage to the cord tissue around the center of the lesion. Moreover, diffuse changes in the expression of chondroitin sulfate proteoglycans (CSPGS) across the whole spinal cord have been described [27]. The procedure was performed by the insertion of a balloon of a Fogarty catheter into the dorsal epidural space (Figure 1C). Since introducing this method, numerous studies have been reported to use identical or slightly modified methods [2,5,7,28,29,30,31,32,33].

As stated above, dorsal displacement of the vertebral body fragments and herniated injured intervertebral disc, compress the anterior surface of the cord causing anterior spinal cord syndrome (Figure 1A,B), which represents around 1–3% of all traumatic SCI [34,35]. However, 50% has also been described [36]. Of the different spinal cord syndromes, anterior cord syndrome has the worst prognosis. Spinal cord infarction, or anterior epidural hematoma, are other, nontraumatic causes [37]. A compressed spinal cord results in the loss of white and gray matter and the formation of surrounded by arachnoiditis and astrogliosis, which interfere in the later stage with axonal regeneration [38]. In a rat spinal cord, localization of the main axonal tracts slightly differs from that in humans. Lesions of the ventrally localized spinothalamic tract induce neuropathic pain, injury of the ventral part of corticospinal tract, impaired locomotor ability and vulnerability of the motor neurons in the ventral horn. As far as we are aware, only a few rat models of ventral spinal cord injury using the sharp blade [39], hypodermic needle [40], inflatable gel [41] or aneurysm compression clip [42] have been proposed. A model with a ventrally placed screw into the cervical vertebra was also introduced to simulate chronic compressive myelopathy in larger animals (cats, dogs) [43,44]. In rat models, methods using progressive compression by a screw or expanding gel have also been developed [45,46]. However, unlike the localized injury induced by clip compression or sharp hemisection, an expanding balloon in the spinal canal spreads the compression force across all laminae of the spinal cord and thus, realistically, simulates the common human SCI.

The overall goal of this study was to develop a reproducible model of the clinically relevant, ventral SCI, and compare it to the well-established model of the balloon-induced dorsal lesion. We used the expandable balloon of the Fogarty catheter to compare the different levels of compression in a rat model of SCI. The evaluation of functional motor and sensory outcome was performed by behavioral testing (Beattie and Bresnahan (BBB) test, ladder rung test and plantar test), tissue loss was assessed by volumetric measurement of white and gray matter in histological cord slices, and the extent of astrogliosis and number of motoneurons were determined using the immunohistochemical antibody markers against Glial fibrillary acidic protein (GFAP+) and choline acetyltransferase (ChAT+).

## 2. Materials and Methods 

### 2.1. Spinal Cord Injury Procedure

**Figure 2:**
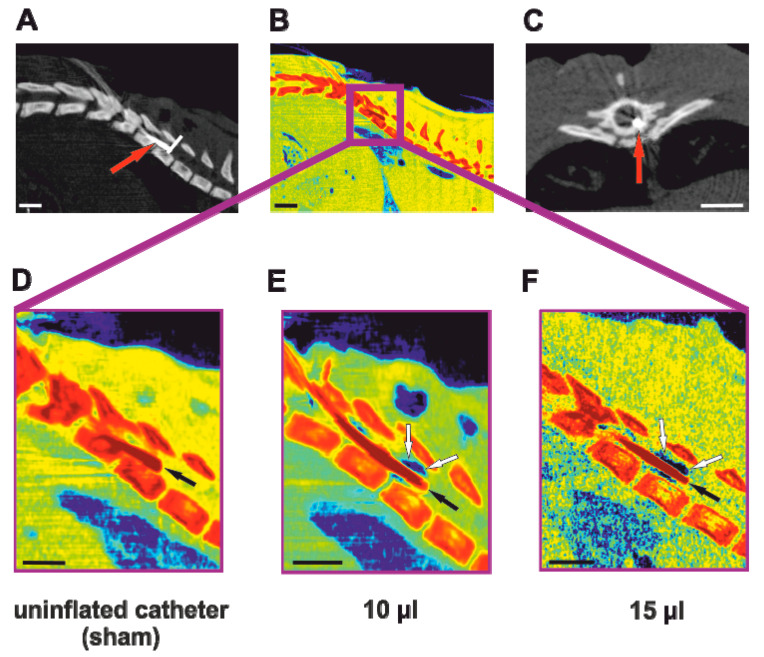
Micro computed tomography (CT) images of ventral epidural catheter placement. In order to evaluate the position of the catheter (red arrows) in front of the spinal cord, micro CT with bone window (**A**,**C**) and soft tissue window (**B**) were applied. Furthermore, detailed images of the deflated balloon (**D**), as well as inflated to 10 µL (**E**) and 15 µL (**F**) volume balloon were obtained. Black arrows show the wire of the catheter, whereas white arrows point to the imaged saline solution indicating the amount of inflated Fogarty´s balloon. Scale bar: 2 mm.

Adult Wistar male rats (*n* = 45) were used as an experimental model. The rats were obtained from the facility breeding center (Institute of Physiology, Academy of Sciences of the Czech Republic, Prague, Czech Republic). The weight of the animals undergoing surgery was 300 ± 30 g and they were 10 weeks old. All the surgery was performed in a specialized operating room for small animals. At the beginning of the procedure, the animals were weighed to optimize anesthesia. General anesthesia was introduced by a face mask with isoflurane (Isoflurane 3.5 vol%, Forane, San Juan, Puerto Rico). Analgesia was applied by intramuscular injection of carprofen (Rimadyl, Cymedica, 4 mg/kg). The animals were perioperatively treated with antibiotics (ATB) prophylaxis by intramuscular injection of gentamicin sulfate (Lek Pharmaceutical, 5 mg/kg). The rat was placed onto a heating pad, which was set for 37 °C to prevent hyper/hypothermia [47]. The back of the animal was shaved and the skin colored with disinfectant. Under sterile conditions, an approximately 2 cm long skin incision between T10 and L2 was performed. The dorsal fascia was cut bilaterally and the spinous processes were exposed with the subsequent removal of processes T10 and T11. A generous laminectomy at the level of T10 was performed using a microronguer, to make enough space for a catheter insertion. A 2-french uninflated Fogarty catheter (Edwards Life Sciences, Irvine, CA, USA) was then carefully introduced from the right lateral aspect of the spinal cord. The right side was chosen as a more comfortable approach for the performing right-handed surgeon. Firstly, epidural adhesions were gently separated with the tip of the catheter to mobilize the spinal cord. After separation of the ventral epidural space, the uninflated catheter was inserted until it reached the desired level of Th8. A midline position of the balloon was maintained by the strictly median position of the down streamed catheter leading to the balloon. This position of the catheter was carefully maintained during the whole procedure. Control images of the catheter position were obtained using the computed tomography (CT) (Figure 2A–F). After a precise placement into the anterior median fissure, the balloon of the catheter was left uninflated (sham group) or rapidly inflated with 10 μL or 15 μL volume of saline for 5 min. A similar procedure was performed in the group with the dorsal lesion, where the catheter was inserted into the dorsal epidural space and inflated with 15 μL of saline. After 5 min the catheter was deflated and carefully removed to prevent further damage of the spinal cord. The separated muscles and incised skin were closed by single nonabsorbable stitches and the wound was treated with a liquid bandage. Throughout surgery, 3.5 vol% isoflurane in air was kept at a flow rate of 0.3 L/minute. In the first days following surgery, the rats were carefully observed for postoperative pain and, whenever necessary, buprenorphine (0.05–0.1 mg/kg, Vetergesic Multidose, Reckitt Benckiser, GB) was administered subcutaneously. For the whole duration of the experiment, the animals were kept at a 12 h light/dark cycle and were allowed to feed and drink ad libitum. The postoperative animals were assisted in urination as required until the neurological functions recovered. 

All the experiments were performed in accordance with the European Communities Council Directive of 22nd of September 2010 (2010/63/EU) regarding the use of animals in research and were approved by the Ethics Committee of the Institute of Experimental Medicine CAS and subsequently by the Section Committee of Czech Academy of Sciences, Prague, Czech Republic (Project No. 54/2017, approved 14th of July 2017). The number of animals was statistically optimized to achieve refinement and reduction.

### 2.2. Behavioral Analysis

#### 2.2.1. Basso, Beattie and Bresnahan (BBB)

The BBB open field test, originally described by Basso, Beattie and Bresnahan [48] was used to assess the locomotor ability of the rats. The rats were placed onto the open-spaced floor, surrounded by boundaries which formed a rectangular shape. The results were evaluated in the range of 0–21 points: 0 indicated a complete lack of motor capability, whereas 21 indicated the best possible score (healthy rat). The measurements were performed weekly for five weeks, starting the first week after SCI.

#### 2.2.2. Ladder Rung

The ladder walking test was used to assess the advanced hindlimb-forelimb coordination of movement. A horizontally placed ladder rung was placed between the start and exit cage. The run was recorded by a high-speed color camera (CamRecord CL600×2, 1280 × 1024 pixel, Stemmer Imaging, Puchheim, Germany) and evaluated using MotoRater 303030 and TSE Motion 8.5.11 software (TSE-systems, Germany). The types of foot or paw placement on the rungs were rated using a seven-category scale (0–6 points) according to their position and errors that occurred in placement accuracy, as previously described by Metz and Whishaw [49]. All the animals were preoperatively trained in the exercise. 

#### 2.2.3. Plantar Analysis

To assess changes in sensory nociceptive pathways, the plantar test was used. The rat was placed into an acrylic box of the standard Ugo Basile test apparatus (Ugo Basile, Comerio, Italy). A mobile infrared heating lamp was then targeted on the plantar surface of the paw, always in the same position. After targeting, a thermal radiant stimulus was applied. Withdrawal latency was automatically measured using a photoelectric-sensitive device. Measurements were performed weekly for five weeks, starting the first week after SCI, with preoperative training. Each paw was stimulated five times. The results were averaged and both paws were pooled together with comparison between groups. Hyperalgesia was determined as an early withdrawal of the paw.

### 2.3. Histological and Immunohistochemical Analysis

After five weeks of behavioral testing, all the animals were intraperitoneally anesthetized with a lethal dose of ketamine (100 mg/kg) and xylazine (20 mg/kg). The comatose animals were transcardially perfused with a phosphate buffer solution (250 mL) and a 4% paraformaldehyde solution in a phosphate buffer (250 mL). The spinal cord was dissected and removed from the spinal column and fixed for another 24 h with 4% paraformaldehyde. After sufficient fixation, 2 cm long spinal cords around the spinal cord lesion were embedded in paraffin wax. Serial cross-sections (5 μm thick, 1 mm interval) were obtained and stained with different staining for further analysis.

#### 2.3.1. Cresyl Violet-Luxol Staining

**Figure 3:**
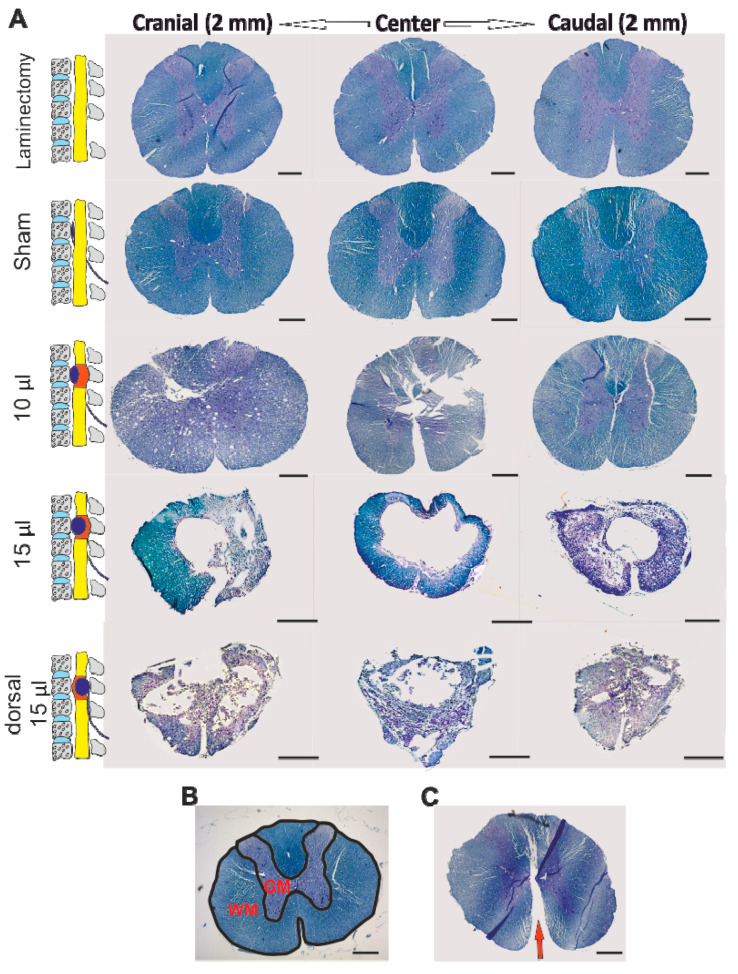
Histological and immunohistochemical assessment of obtained cross sections. Graded spinal cord lesion in and around the epicentre (spinal level Th8) correlated with the level of compression (**A**). Morphometry of the white and grey matter according to the histological images was performed in an area of 7 mm cranially and caudally to the lesion centre (**B**). In the group of 10 μL, splitting of the anterior median fissure was observed—red arrow points towards the split anterior median fissure (**C**). Scale bar: 500 μm.

For visualization of white and gray matter, and further morphometric analysis, Cresyl violet and Luxol fast blue staining were used (Figure 3A). From each group, five animals were obtained. A total number of fifteen cross-sections, including the center of the lesion and areas both cranially and caudally, were observed and photographed with an Axioskop 2 plus microscope (Zeiss, Oberkochen, Germany). The acquired images were evaluated for the total spared area of gray and white matter by ImageJ software (NIH, Bethesda, MD, USA) (Figure 3B).

#### 2.3.2. GFAP Staining

**Figure 4:**
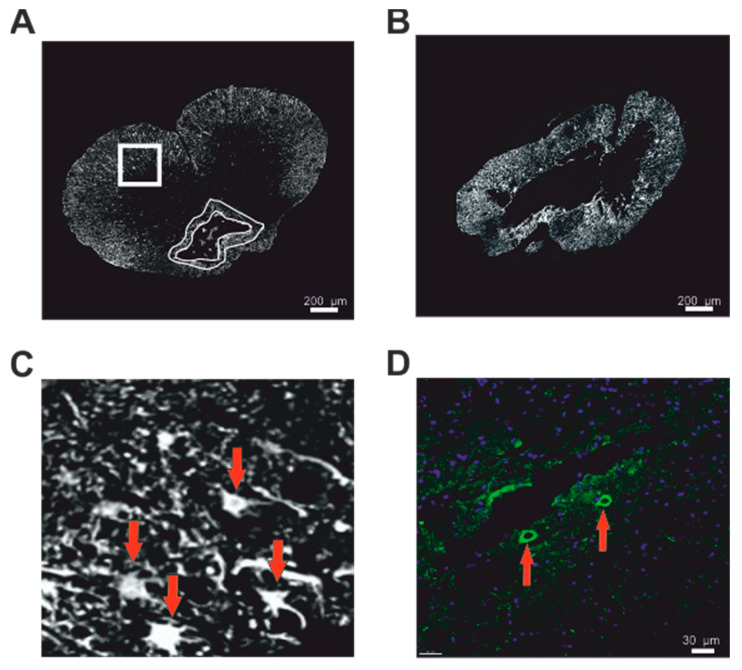
(**A**—4 mm cranially from the lesion epicentre, **B**—lesion epicentre at Th8) Volumetric measurement of the astrogliosis around the central cavity was performed in the GFAP stained immunohistochemical images. (**C**) Detailed view with red arrows pointing to protoplasmic astrocytes in GFAP staining. (**D**) The impact of the lesion on the survival of motoneurons was evaluated using the ChAT+/DAPI immunostaining: red arrows show the motoneurons.

Immunohistochemical visualization of the astrogliosis and protoplasmic astrocytes was obtained by staining with CY3-conjugated primary antibody against GFAP (1:400, Sigma-Aldrich, St. Louis, MO, USA) (Figure 4A,B). From each group, five animals were obtained. A total number of ten cross-sections, including the center of the lesion and areas both cranially and caudally, were observed and photographed with an Axioskop 2 plus microscope (Zeiss, Oberkochen, Germany). Acquired images were evaluated for the total area of GFAP high intensity signal around the malatic cavity representing the astrogliosis by ImageJ software (NIH, Bethesda, MD, USA). On the same images, the total number of protoplasmic astrocytes was manually counted (Figure 4C). 

#### 2.3.3. Motoneurons

Immunohistochemical labelling of motoneurons with choline acetyltransferase (ChAT) antibody (1:75, NBP1-30052) was used to assess the surviving number of cells (Figure 4D), which were counted in five cross sections. From each group, five animals were obtained. The counted motoneurons were averaged and compared between the groups. 

### 2.4. Statistics

To analyze the effect of each separate group of animals after ventral SCI, different statistical tests were used. The two-way repeated measurement ANOVA test was applied for the BBB test, plantar test, areal measuring of astrogliosis, white/gray matter sparing, astrocyte and motoneuron number. The ladder rung test was assessed using the one-way ANOVA test. The Student–Newman–Keuls (SNK) post hoc pair-to-pair test was used to specify for which groups, and at which timepoints, the changes were significant (all in Sigmastat 3.1, Sistat Software Inc., San Jose, CA, USA). The differences were considered statistically significant if *p* < 0.05.

All the presented data in graphs were expressed as arithmetical means, with the standard error of the mean included. A significance in the text or in the graph is marked as follows: * *p* < 0.05, ** *p* < 0.01, *** *p* < 0.001. *p* Values along with the q values, are displayed in the Supplementary Materials.

## 3. Results

### 3.1. BBB Test

**Figure 5:**
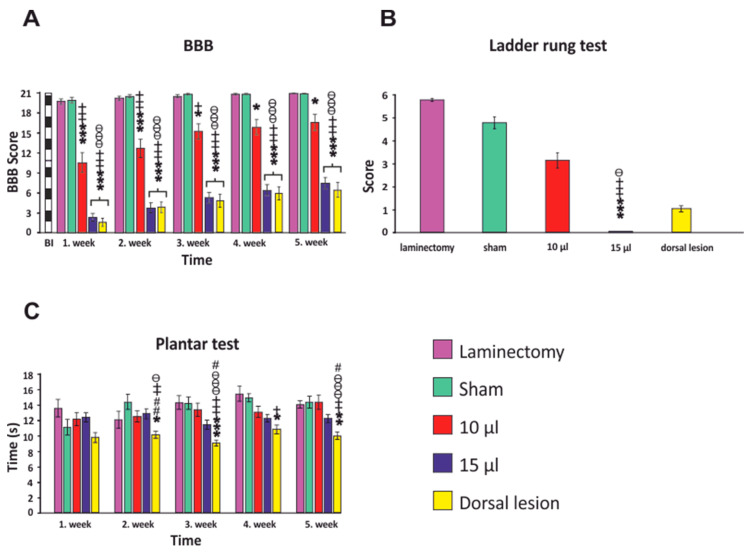
Behavioral assessment of the SCI rats. BBB open field test revealed significant differences between groups. The inflated balloon with 15 µL both ventrally and dorsally resulted in severe paraparesis with only mild improvement (**A**). Ladder rung score at the fifth week showed a reduced performance in both lesioned groups, where animals in the 15 µL group were unable to cross the ladder (**B**). Evaluation of the plantar test indicated significant hyperalgesia in the group with the dorsal lesion (**C**). For statistical details, see Tables S1–S3 in the Supplementary Material. Data are expressed as mean ± SEM. * *p* < 0.05 versus laminectomy; ** *p* < 0.01 versus laminectomy; *** *p* < 0.001 versus laminectomy; + *p* < 0.05 versus sham; ++ *p* < 0.01 versus sham; +++ *p* < 0.001 versus sham; ϴ *p* < 0.05 versus 10 µL; ϴϴ *p* < 0.01 versus 10 µL; ϴϴϴ *p* < 0.001 versus 10 µL; # *p* < 0.05 versus 15 µL.

Basic hind limb recovery was assessed using the BBB locomotor open field testing, which was performed weekly after SCI. Following surgery, no significant changes between the simple laminectomy and sham groups were observed. The BBB scores in those groups varied from 19.75 (±0.39) one week after SCI, to 21 (±0.05) at the end of the experiment. In the group with 10 µL SCI, a significant impairment of locomotor function was observed. The first week after SCI, the mean score of the animals was 10.58 (±1.48) points, which was significantly different from both the unlesioned groups and 15 µL group (*p* < 0.001). Throughout the duration of the experiment, motor functions gradually improved, finishing with a mean BBB score 16.62 (±1.17) after five weeks, which was still significantly different when compared to the 15 µL group (*p* < 0.001). In the 15 µL SCI group, which had a more severe deficit, the mean score was 2.33 (±0.6) in the first week after SCI, which was significantly different when compared to all the other groups (*p* < 0.001). A mild improvement of motor functions occurred over the subsequent four weeks with a final score of 7.48 (±0.85). The group with the dorsal lesion achieved similar results to the 15 µL SCI group with the final score in the fifth week of 6.44 (Figure 5A). No significant difference between the right and left leg was observed, so the results from both legs were pooled together.

### 3.2. Ladder Rung Test

Advanced coordinated locomotor functions with precise paw placement were tested, using the ladder walking test, the fifth week after injury. From the uninjured groups, the animals in the laminectomy group achieved a score of 5.75 (±0.07), and in the sham group 4.76 (±0.25). No significant difference between those groups was observed. The rats in the 10 µL group were able to cross the ladder with some missteps and achieved a score of 3.12 (±0.33). The animals in the 15 µL group were not able to cross the ladder and were scored with 0 points, which was significantly different from both the uninjured groups and 10 µL group (*p* < 0.001, *p* < 0.05). The rats in the dorsal group were occasionally able to cross the ladder, which resulted in the final score of 1 (±0.13) (Figure 5B).

### 3.3. Plantar Test

An assessment of the nociceptive spinothalamic tract was performed weekly following SCI using the plantar test. No differences were observed between the left and right paw, so the results from both legs were pooled together. The rats in the group of the dorsal lesion displayed a significant hypersensitive reaction starting the second week after injury compared to the uninjured and ventral lesion groups, indicating the lower threshold to nociceptive stimulus (hyperalgesia). In the 15 µL ventral lesion group, especially in third to fifth week, a trend was observed towards the hypersensitive reaction, but this didn’t reach significance. The final withdrawal times of the rats were as follows: laminectomy—13.7 s, sham—14.6 s, 10 µL—14.5 s, 15 µL—11.6 s, dorsal lesion—9.9 s. The results are compatible with the hypothesized greater effect of the dorsal lesion on the spinothalamic tract (Figure 5C).

### 3.4. White and Grey Matter Sparing

The morphometric measurement of the white and grey matter to evaluate the sparing of the spinal cord tissue was performed on 15 axial histological slices (seven sections cranially and caudally to the center of the lesion, which was determined as the section with the smallest area of residual spinal cord tissue).

**Figure 6:**
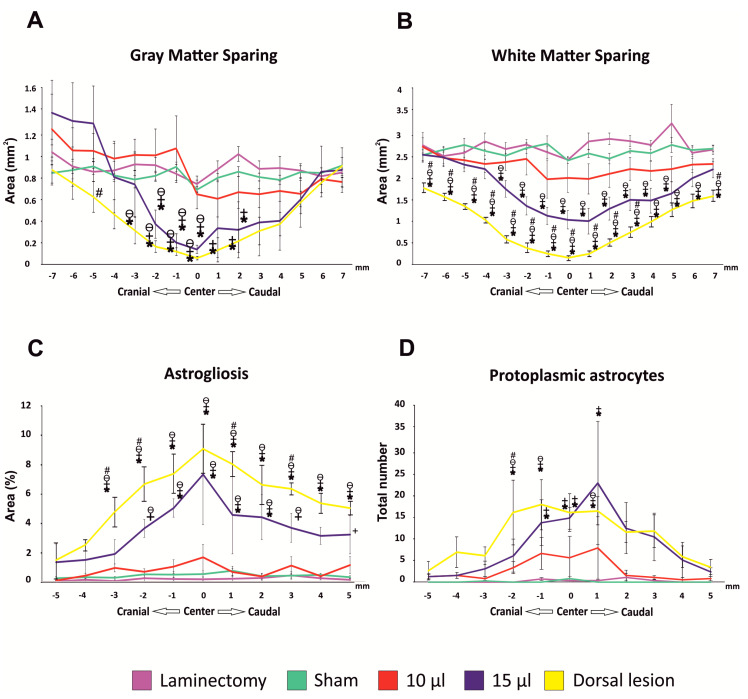
Histological and immunohistochemical assessment. Volumetric measurement of both grey (**A**) and white (**B**) matter revealed significantly decreased total spared areas in both 15 μL groups (dorsal and ventral). White matter was also significantly decreased in the 10 μL group. Astrogliosis was observed in the most injured group, and in the center of the lesion covered around 7% of the whole cross section (**C**). Extent of the astrogliosis correlated with the significantly increased number of protoplasmic astrocytes (**D**). For statistical details, see Tables S4–S7 in the Supplementary Material. Data are expressed as mean ± SEM. * *p* < 0.05 versus laminectomy; + *p* < 0.05 versus sham; ϴ *p* < 0.05 versus 10 µL; # *p* < 0.05 versus 15 µL.

A decreased area of spared grey matter was observed in the 15 µL group, with significant differences around the center of the lesion both cranially and caudally. The same volume of saline in the inflated balloon, placed dorsally to the spinal cord, resulted in a significantly increased loss of grey matte, compared to the ventral lesion (Figure 6A). 

The morphometry of the white matter revealed significantly impaired sparing in both the SCI groups of 15 µL and 10 µL when compared to the uninjured groups. Moreover, the animals in the 15 µL group also had a significantly lower spared area of white matter when compared to the animals in the 10 µL group. Similar to the grey matter, a dorsally placed balloon caused significantly greater damage to the white matter than in the ventrally placed groups (Figure 6B).

### 3.5. Astrogliosis) and Number of Protoplasmic Astrocytes

The morphometric measurement of the GFAP-positive area, representing the astrogliosis around the main cavity in the immunohistochemical staining, was performed on 11 axial slices (five sections cranially and caudally to the center of the lesion, which was determined as the section with the smallest area of the residual spinal cord tissue). In both the uninjured groups and the 10 µL group, astrogliosis was below 1% of the total area of the cross section. A significant increase of the astrogliosis was observed in the 15 µL group, with the peak in the center of the lesion (7.2 ± 3.4%). An even greater GFAP-positive area was observed in the dorsal lesion group, with averaged astrogliosis of 8.9% around the center of the lesion (Figure 6C). 

The absolute number of protoplasmic astrocytes (PA) around the astrogliosis was counted in the same GFAP-positive immunohistochemical axial slices. In the uninjured groups, sporadic PAs were found. Elevated numbers of PAs were found in both the 10 µL and 15 µL groups. In the 15 µL group, there was a significantly higher number of astrocytes with the peak appearance around the center of the lesion. The dorsal lesion groups achieved similar results to the 15 µL group (Figure 6D). 

### 3.6. Motoneurons

**Figure 7:**
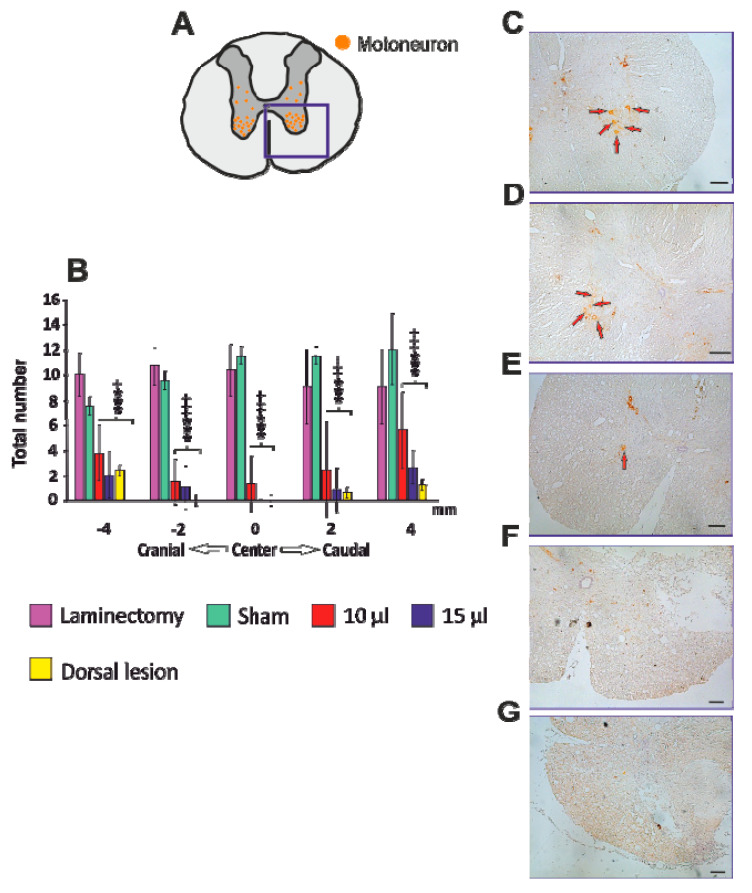
Immunohistochemical assessment of motoneurons. Total number of motoneurons localized in the ventral horn was counted on axial slices around the center of the lesion (**A**). Total number of motoneurons was significantly decreased in both ventrally injured groups and in dorsal SCI across the lesioned area (**B**). Representative immunohistochemical images with visible motoneurons (red arrows) from each group are shown (**C**—laminectomy, **D**—sham, **E**—10 μL, **F**—15 μL, G—dorsal 15 μL). For statistical details, see Table S8 in the Supplementaty Material. Scale bar: 200 μm.Data are expressed as mean ± SEM. * *p* < 0.05 versus laminectomy; ** *p* < 0.01 versus laminectomy; *** *p* < 0.001 versus laminectomy; + *p* < 0.05 versus sham; ++ *p* < 0.01 versus sham; +++ *p* < 0.001 versus sham

To assess the impact of the lesion on the survival of the motoneurons, the total number of ChAT-positive neurons were counted on five axial slices (two sections cranially and caudally to the center of the lesion, which was determined as the section with the smallest area of the residual spinal cord tissue). A significantly decreased number of motoneurons was found in all the SCI groups, 15 µL dorsal lesion, 15 µL and 10 µL ventral groups, when compared to the uninjured groups. The average number of motoneurons in the SCI groups varied from 0 to 5.9, whereas in the uninjured groups it was 9 to 21. No differences between the sham and laminectomy groups were found (Figure 7B).

## 4. Discussion

For successful translation, appropriate animal models of SCI are needed. In this study, we introduced a novel and reproducible experimental model of a balloon-induced, ventral ischemic-compression thoracic spinal cord injury in the rat. Both the behavioral results and histochemical analysis show that the anterior balloon inflation induces a volume-dependent injury, preferably in the ventral spinal cord.

It was previously shown that a contusion model using epidural dorsal placement of the 2-french Fogarty catheter is a safe and easily reproducible model of SCI [26]. The same approach to the spinal canal was successfully utilized in this study. Anterior placement of the catheter to the final position on the anterior side of the spinal cord requires a meticulous surgical technique. The challenging moment is removal of the catheter from the epidural space. The end of the catheter leading to the balloon is secured in the midline position to ensure positioning of the balloon. Subsequently, the catheter has to leave the epidural space in the acute angle preventing an undesired movement of the distal tip, which needs to be secured by the surgeon. Even though an uninflated balloon is inserted into the anterior median fissure, inflation with a larger amount of saline (10, 15 μL) probably causes minor deviation from the midline in some cases (Figure 2C). However, these small malpositions didn’t result in any significant interlimb differences. A similar method of catheter placement could be used for the creation of the ventrolateral quadrant lesion, as an equivalent to the dorsolateral quadrant lesion [50]. However, this model would require further experiments and standardization. To avoid causing damage to the spinal cord during the introduction of the catheter, we recommend the use of an operative microscope or loupe glasses. We compared different degrees of ventral spinal cord compression (10 μL group and 15 μL group) to the unlesioned animals (laminectomy approach group and anterior catheter placement group) and 15 μL dorsal lesion with histological (white and gray tissue sparing), immunohistochemical (astrogliosis formation, protoplasmic astrocyte and motoneuron count) and functional outcome (BBB, ladder rung, plantar test) evaluations. The safety of the catheter placement to the anterior epidural space was demonstrated, when there was no significant impairment of the motor or sensory functions, together with no significant tissue loss and no identifiable area of astrogliosis observed.

In order to obtain a moderate to severe spinal cord injury, we chose to compare 10 and 15 μL inflation of the Fogarty catheter. In the 10 μL group, initial loss of muscle strength, paw placement mechanics and coordination of the limbs, with preserved body-weight bearing, were observed in the BBB test. Throughout the duration of the experiment, these functions were mostly restored with a final clinical image of mild paraparesis, leading to an increased number of missteps in the ladder rung test. A mild tissue loss, caudally from the injury level, resulted in a significant difference in the total white matter area. Despite no observable changes in astrogliosis or protoplasmic astrocytes, a significant decrease in the number of motoneurons was observed. Spinal cord compression with 15 μL, resulted in a severe behavioral deficit and mostly complete paraplegia in the first week after SCI. Some spontaneous recovery of the motor functions was observed but the final result of 7.5 points in the BBB test indicated that no weight support was restored. These highly disabled rats were unable to cross the ladder and had a mild insignificant increase of lower limb hyperalgesia. The severe neurodeficit correlated with significantly decreased grey and white tissue sparring, increased astrogliosis formation with a number of protoplasmic astrocytes and a decreased number of motoneurons. To evaluate the different effect of the ventrally placed catheter, we compared all groups with the traditional method of the dorsal balloon-induced SCI. Generally, a similar volume of ventrally inflated balloon in injured rats displayed slightly worse motor functions, resulting in a declined ladder walking test, and a significantly higher threshold to nociceptive stimuli, indicating less damage to the spinothalamic tract. Interestingly, the histological and immunohistochemical evaluation revealed significantly greater damage to the spinal cord in the dorsally lesioned rats. Rats in the ventrally and dorsally injured groups displayed difficulties with urination. However, rats in the 10 μL group spontaneously recovered within a few days after SCI. Animals in the 15 μL group and dorsal lesioned group required assisted voiding during 2–3 weeks post SCI.

Overall, our functional motor results are comparable to those obtained by dorsal compression by Vanicky et al. [26]. Notably, a number of our previous studies, where the model of dorsal 15 μL compression in untreated control rats was used, consistently reported slightly more severe injuries (approx. one to two BBB points lower), similar to this study [29,51,52,53,54]. This slight difference may be due to indirect compression of the cortico-spinal motor tract, which in rats is mainly localized in the dorsal region of the spinal cord and thus more affected by the dorsal compression. However, despite lower scores in the BBB test, the dorsally injured rats achieved better scores in the fine movement required for the ladder walking test. According to our histological images, it was also suggested that compression was in some cases influenced by anterior median fissure splitting. The median anterior placement of the balloon often resulted in splitting of the cord, enabling tissue shifting with softening of the compression. Occasional splitting of the cord was even more obvious in the 10-μL group, which also resulted in major inhomogeneity in this group (Figure 3C). With an increased volume in the spinal canal, the negative consequence of this phenomenon diminished with quite consistent severe injury in the 15-μL group.

In the light of clinical relevancy, compression and contusion injuries of SCI are probably the most suitable models [55,56]. SCI in humans is often the result of a high-energy traumatic impact on the spinal column, causing vertebral fracture with the dislocation of fragments. Contusion injuries are introduced by various electromagnetic or weight dropping impactors [25,57]. The major challenges of this model are in variability of the functional recovery in the post-injury period [58,59]. Consistency in improvement of the injury can be achieved by a digitalized computer-controlled impactor, together with stabilization of the rat’s body and spine [60].

Compression models of SCI can either be achieved by balloon techniques, by clipping of the cord or with impactors and weight drop. Chronic compression injuries modeling cervical myelopathy have also been developed [41]. In the clip injury models, first described in 1978 [61], calibrated clips were used to exert a force of 35–50 g. However, clip compression delivering the force from both lateral sides does not represent a common mechanism of human SCI. A balloon-induced acute compression of the dorsal spinal cord is widely used in rats, dogs, rabbits or primates [26,62,63,64]. Anterior compression injury in cats was also achieved by an adjustable screw implanted through the vertebral body [43,45,65] or by an implanted expandable compression device [66]. Slow tightening of the screw is more suitable for chronic compression studies. Notably, technical difficulties during surgery to obtain the correct position of the screw are not negligible. Inflation of the balloon can be performed rapidly in a few seconds to simulate acute injury, or graded in a subacute manner. In particular, chronic compressive myelopathy can also be achieved by securing the catheter in situ with a subsequent slow inflation of the balloon over the weeks. Interestingly, rapid balloon inflation carries a higher risk of neurogenic pulmonary edema [67].

In our study we described a model of anterior spinal cord compression injury, matching the predominant type of injury commonly seen in humans. Using a well-established and relatively noncomplicated approach, this method is easily reproducible and enables lesions of different severity.

## Supplementary Materials

The following are available online at https://www.mdpi.com/2227-9059/8/11/477/s1, Table S1: BBB test statistics, Table S2: Ladder rung test statistics, Table S3: Plantar test statistics, Table S4: Gray matter sparing statistics, Table S5: White mater sparing statistics, Table S6: Astrogliosis statistics, Table S7: Protoplasmatic astrocytes statistics, Table S8: Motoneurons statistics.
