# Supplementary figures and images for: New Model of Ventral Spinal Cord Lesion Induced by Balloon Compression in Rats
Source: Biomedicines. 2020 Nov 5;8(11):477. doi: 10.3390/biomedicines8110477 (PMC7694490; doi:10.3390/biomedicines8110477)

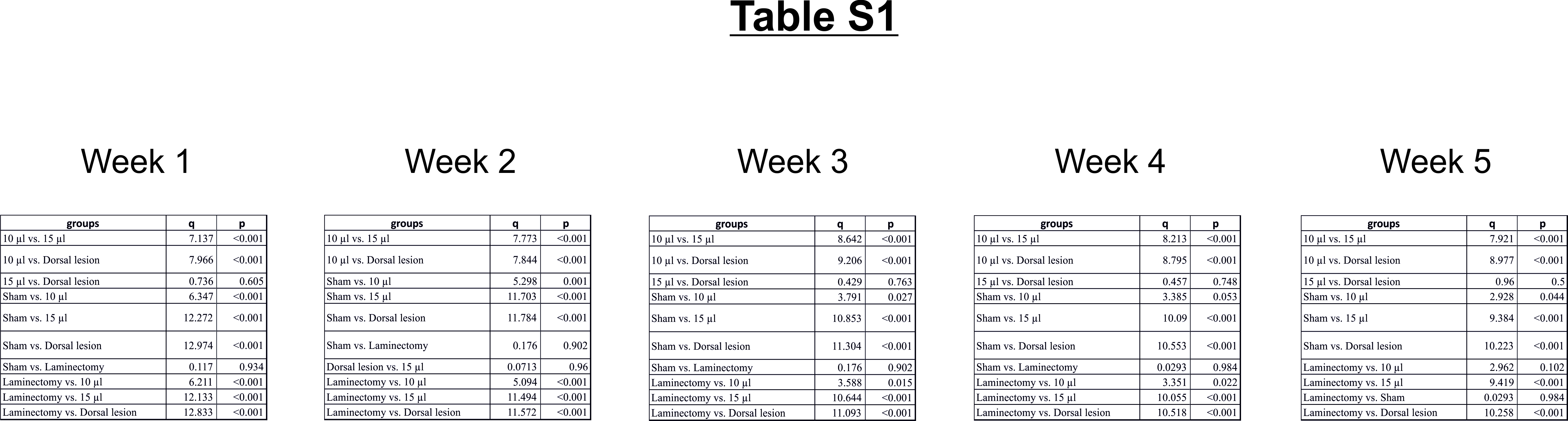

Supplement: Supplementary file 1 [file biomedicines-08-00477-s001.zip › Table S1.jpg]

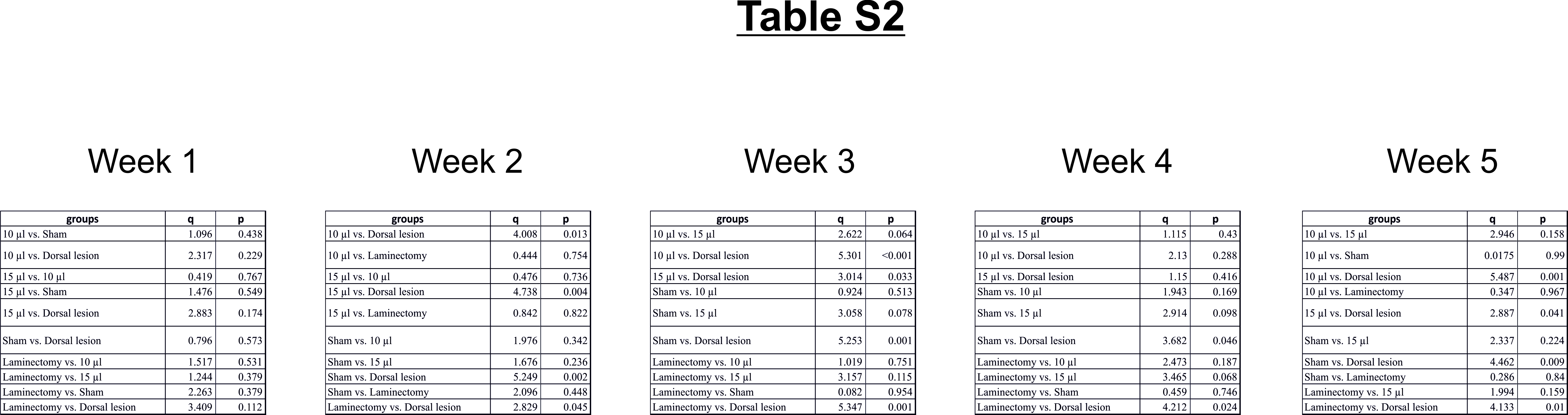

Supplement: Supplementary file 1 [file biomedicines-08-00477-s001.zip › Table S2.jpg]

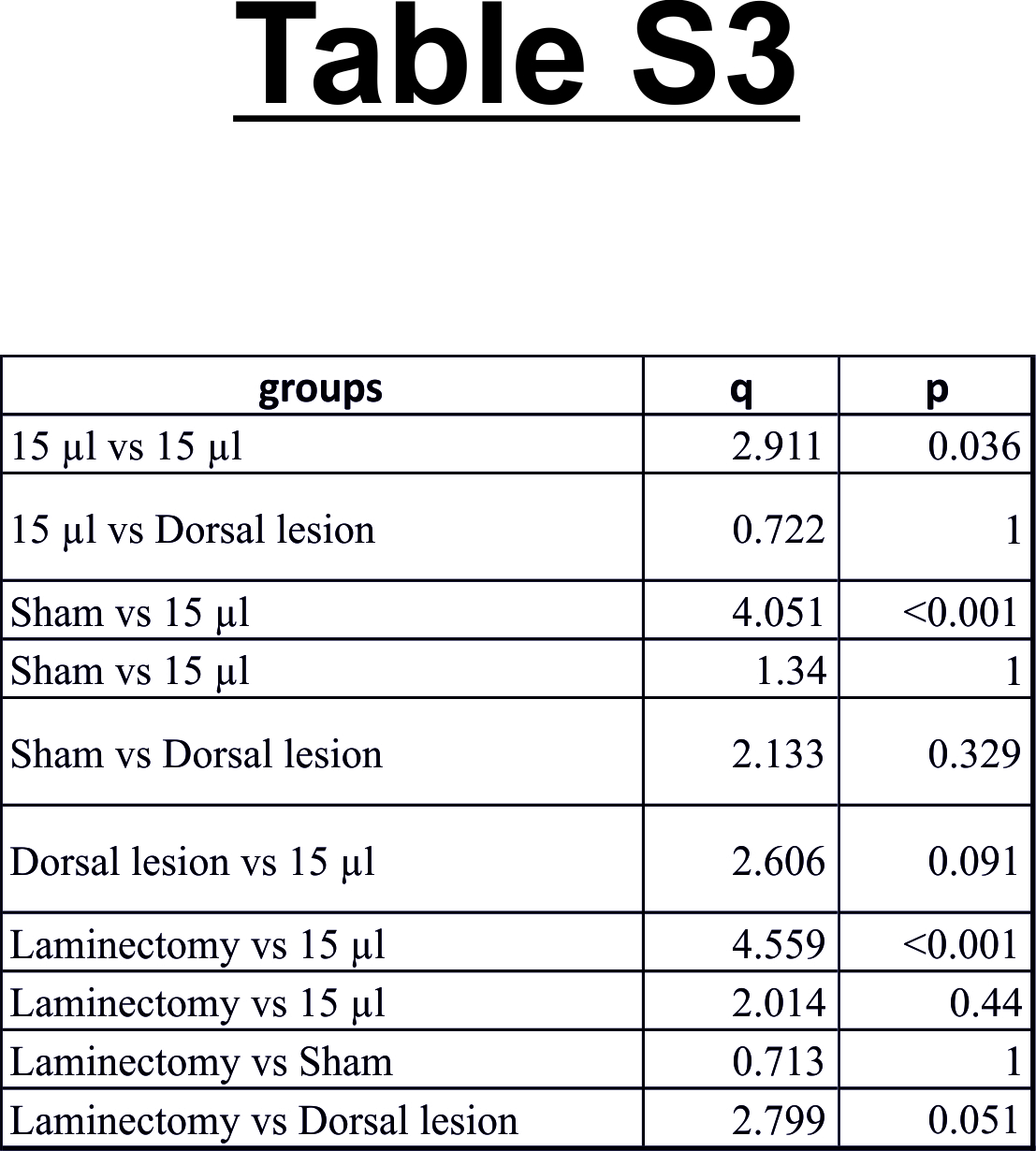

Supplement: Supplementary file 1 [file biomedicines-08-00477-s001.zip › Table S3.jpg]

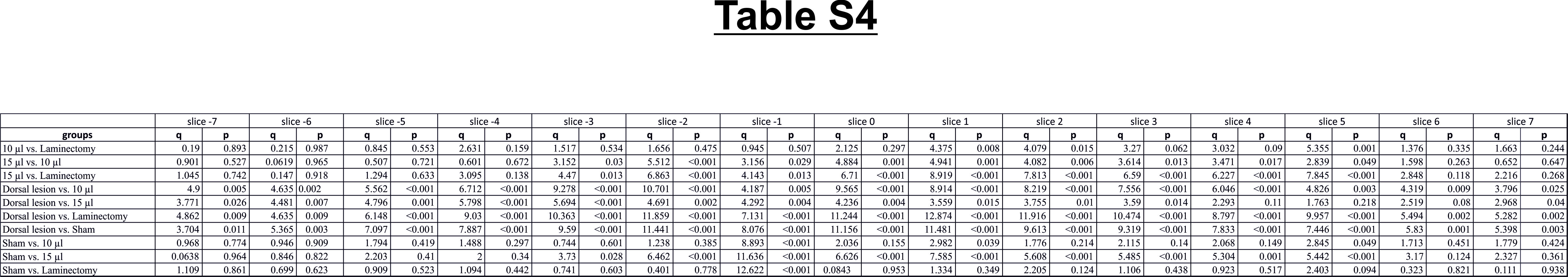

Supplement: Supplementary file 1 [file biomedicines-08-00477-s001.zip › Table S4.jpg]

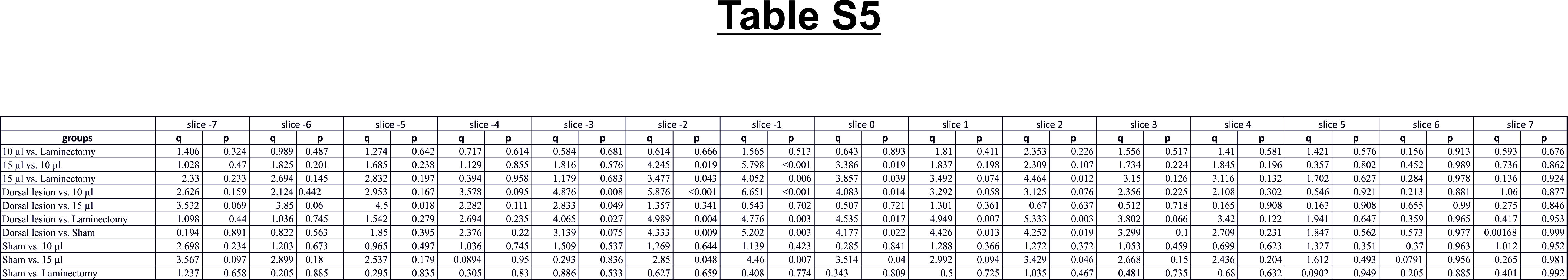

Supplement: Supplementary file 1 [file biomedicines-08-00477-s001.zip › Table S5.jpg]

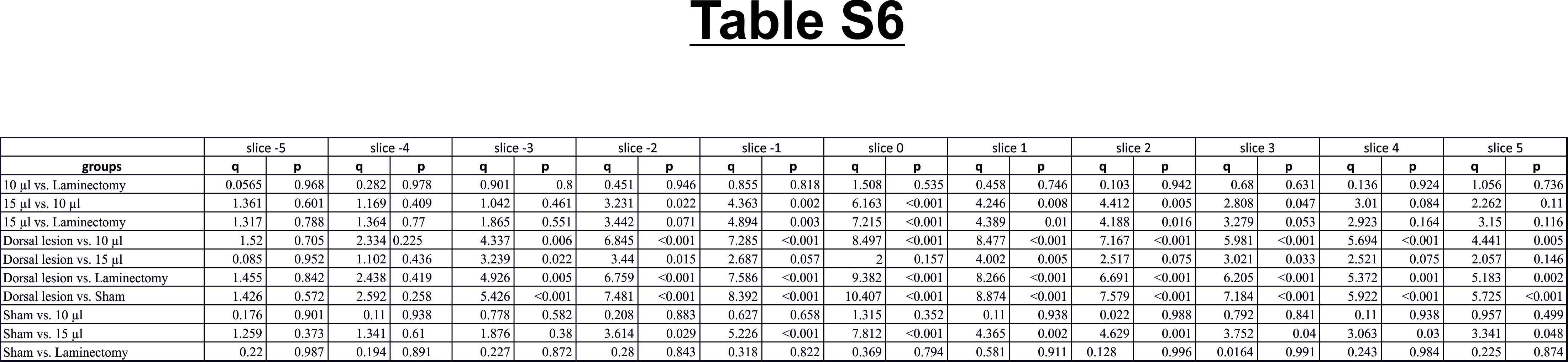

Supplement: Supplementary file 1 [file biomedicines-08-00477-s001.zip › Table S6.jpg]

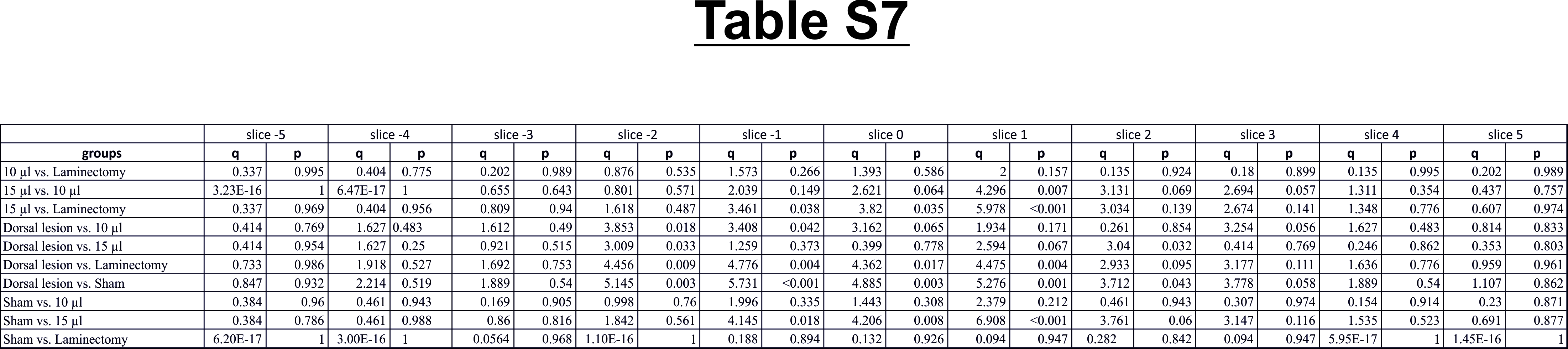

Supplement: Supplementary file 1 [file biomedicines-08-00477-s001.zip › Table S7.jpg]

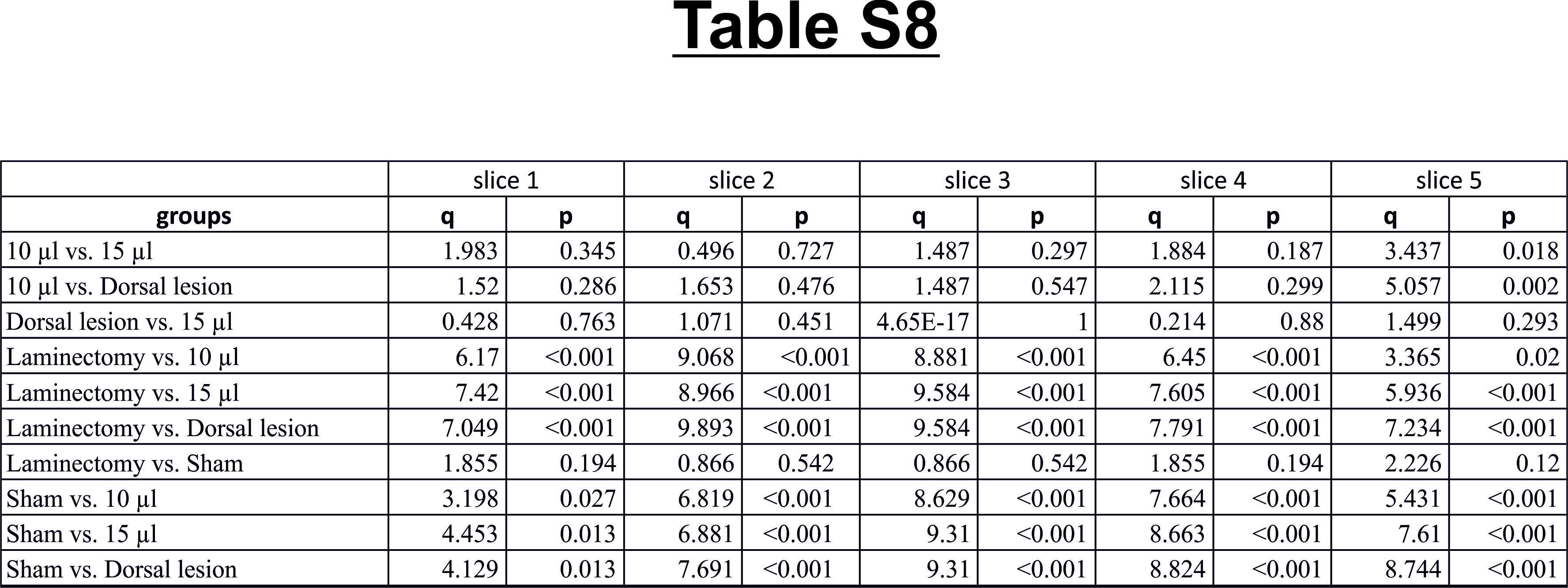

Supplement: Supplementary file 1 [file biomedicines-08-00477-s001.zip › Table S8.jpg]
